# College and Hospital Notes

**Published:** 1911-01

**Authors:** 


### COLLEGE AND HOSPITAL NOTES

THE Riverside Hospital at No. 113 Lafayette Avenue has passed from the list of Buffalo institutions and a new and larger hospital is to be erected by the Lafayette General Hospital Association, located on the same site, which will take its place. The certificate of incorporation has been filed with the county clerk.

The new association is composed of a number of physicians who have bought the property on Lafayette Avenue and have begun improvements to the building. A new heating plant has been installed and new operating rooms have been planned. General renovation will soon take place with a view to make the hospital an efficient and modern institution, where busy practitioners will find everything at hand at any time of the day or night. Extensive plans for its development and increasing its sphere of usefulness in the immediate future are under consideration by the management.

The institution will be conducted as a non-sectarian general hospital, where all reputable physicians and surgeons will be welcome. It is no longer a private enterprise but will be governed by a board of trustees, of whom the executive officers are: Dr. H. C. Rooth, president; Dr. J. H. Potter, vice-president, and Dr. Hugh McIntyre, secretary and treasurer.

THE Buffalo General Hospital has established a social service department and Miss Lucy Stockton has been placed in charge. She has made extended observation of the methods in vogue at the Massachusetts General Hospital in conducting a similar service, hence brings to the work here a valuable experience.

GYNECOLOGICAL Clinics, from November 1, 1910, to May 1, 1911, are held by Dr. Ralph Waldo, at the Lebanon Hospital, New York, on Thursdays, beginning at 3 o'clock.

THE Barnard Free Skin and Cancer Hospital, Saint Louis, has completed its new hospital building and it was opened for inspection December 20, 1910. The JOURNAL acknowledges an invitation from the Board of Directors to attend the ceremonies, for which courtesy its best thanks are returned.

